# Genome-Wide Association Study of African and European Americans Implicates Multiple Shared and Ethnic Specific Loci in Sarcoidosis Susceptibility

**DOI:** 10.1371/journal.pone.0043907

**Published:** 2012-08-27

**Authors:** Indra Adrianto, Chee Paul Lin, Jessica J. Hale, Albert M. Levin, Indrani Datta, Ryan Parker, Adam Adler, Jennifer A. Kelly, Kenneth M. Kaufman, Christopher J. Lessard, Kathy L. Moser, Robert P. Kimberly, John B. Harley, Michael C. Iannuzzi, Benjamin A. Rybicki, Courtney G. Montgomery

**Affiliations:** 1 Arthritis and Clinical Immunology Research Program, Oklahoma Medical Research Foundation, Oklahoma City, Oklahoma, United States of America; 2 Department of Public Health Sciences, Henry Ford Health System, Detroit, Michigan, United States of America; 3 Division of Rheumatology, Cincinnati Children’s Hospital Medical Center, Cincinnati, Ohio, United States of America; 4 The United States Department of Veterans Affairs Medical Center, Cincinnati, Ohio, United States of America; 5 Department of Pathology, University of Oklahoma Health Sciences Center, Oklahoma City, Oklahoma, United States of America; 6 Department of Medicine, University of Alabama at Birmingham, Birmingham, Alabama, United States of America; 7 Department of Medicine, SUNY Upstate Medical University, Syracuse, New York, United States of America; Peninsula College of Medicine and Dentistry, United Kingdom

## Abstract

Sarcoidosis is a systemic inflammatory disease characterized by the formation of granulomas in affected organs. Genome-wide association studies (GWASs) of this disease have been conducted only in European population. We present the first sarcoidosis GWAS in African Americans (AAs, 818 cases and 1,088 related controls) followed by replication in independent sets of AAs (455 cases and 557 controls) and European Americans (EAs, 442 cases and 2,284 controls). We evaluated >6 million SNPs either genotyped using the Illumina Omni1-Quad array or imputed from the 1000 Genomes Project data. We identified a novel sarcoidosis-associated locus, *NOTCH4*, that reached genome-wide significance in the combined AA samples (rs715299, *P*_AA-meta_ = 6.51×10^−10^) and demonstrated the independence of this locus from others in the MHC region in the same sample. We replicated previous European GWAS associations within *HLA-DRA, HLA-DRB5, HLA-DRB1*, *BTNL2,* and *ANXA11* in both our AA and EA datasets. We also confirmed significant associations to the previously reported *HLA-C* and *HLA-B* regions in the EA but not AA samples. We further identified suggestive associations with several other genes previously reported in lung or inflammatory diseases.

## Introduction

Sarcoidosis is a systemic disease characterized by granulomatous inflammation that primarily affects the lungs, but can affect any organ [1], [2], [3]. While the etiology of this disease remains elusive, the pathophysiology likely involves a dysregulated immune response to environmental agents in a genetically susceptible host. Several environmental exposures have been associated with sarcoidosis including mold, inorganic particles, and insecticides [4], [5], [6]. A significant genetic component to sarcoidosis susceptibility is supported by a 2.5 fold elevated disease risk in siblings and parents of cases [7] as well as potential disease susceptibility loci identified from both linkage and association studies [8], [9], [10], [11], [12].

Sarcoidosis impacts individuals of all races, ages and genders [13], but in the U.S. is most frequent in AAs [14], [15], with disease onset peaking between the ages of 20 and 39 years [16]. The AA population is more commonly affected than EAs [16], [17], [18], [19], with a three-fold higher lifetime risk (2.4%) and age-adjusted annual incidence (35.5 per 100,000) compared to EAs (0.85% and 10.9 per 100,000, respectively). AA patients have higher disease severity and more extra-thoracic involvement than EA patients and are less likely to have disease that resolves [20]. Ethnicity specific prevalence and severity support the involvement of genes and further suggest ethnicity-specific genetic risk profiles.

Genetic associations with specific HLA alleles and sarcoidosis have repeatedly been reported [21], [22], [23], [24]. Heterogeneity of these HLA effects in sarcoidosis across ancestries was observed in the ACCESS study [23] suggesting that while the HLA-DRB1*1101 allele was associated with sarcoidosis in AAs and EAs, the HLA-DRB1*1501 allele was associated with sarcoidosis only in EAs [23]. Recent studies have reported additional susceptibility loci including *BTNL2* [9], [25], [26] in both EAs and AAs, and *ANXA11* [11] and *RAB23* [27] in Germans. The first genome-wide linkage study of AA sarcoidosis families performed by our group found prominent linkage signals on chromosome 5, at 5q11.2, 5p13, and 5q31 [10]. Our admixture study confirmed the latter two of these effects and found regions on chromosomes 6p22.3 and 17p13.3–17p13.1 associated with increased African ancestry [28]. Based on clear evidence of the involvement of genes in the onset and manifestation of sarcoidosis, we sought to confirm sarcoidosis genetic risk loci reported in association scans of European populations and to identify novel risk loci by conducting the first genome-wide association study (GWAS) of sarcoidosis in an American population. We present results from a family-based discovery cohort of AAs as well as two independent replication sets of AA cases and controls and EA cases and controls.

## Results

### Genome-wide Association Scan of AA Discovery Set

**Figure 1:**
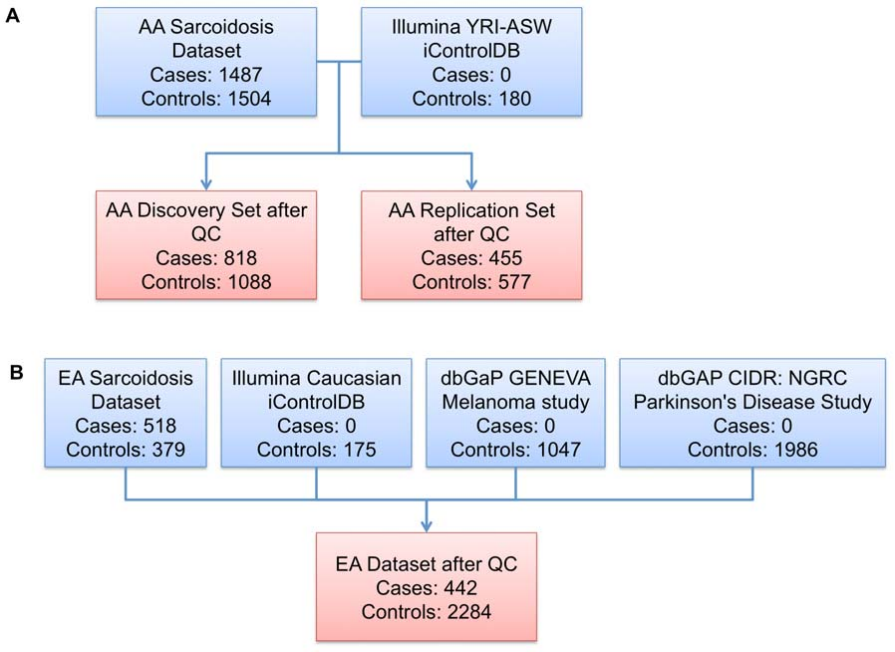
A graphical overview of the GWAS datasets. (A–B) Summary of the AA (A) and EA (B) datasets.

**Figure 2:**
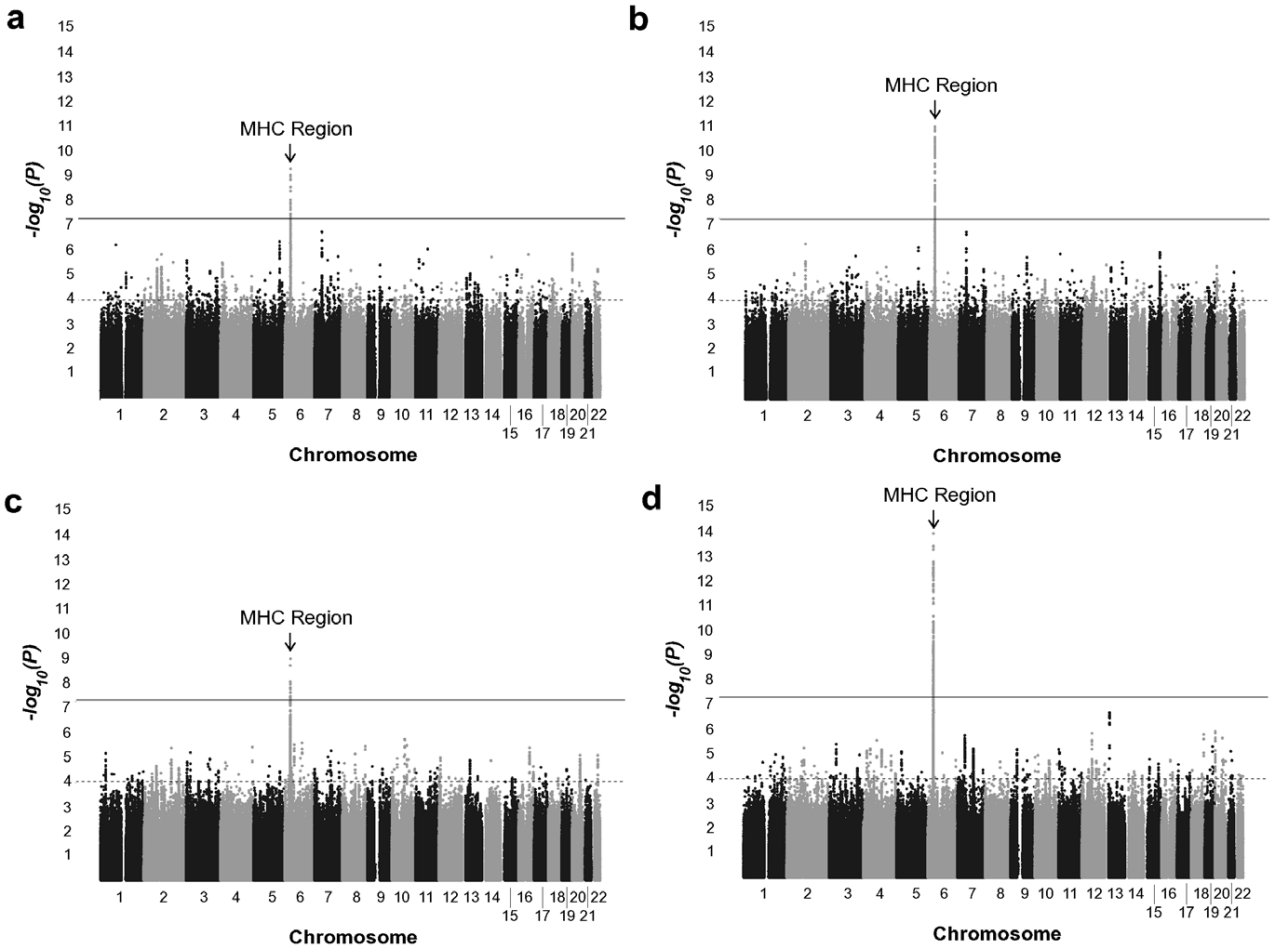
Manhattan plots of SNP-sarcoidosis association test results. (A–D) Association results in the AA discovery set (A), a meta-analysis between the AA discovery and AA replication sets (B), the EA dataset (C), and a meta-analysis of the AA discovery, AA replication and EA datasets (D). The black horizontal line represents the threshold for genome-wide significance (*P*<5×10^−8^) and the gray line is the suggestive evidence of association threshold (*P*<1×10^−4^).

**Figure 3:**
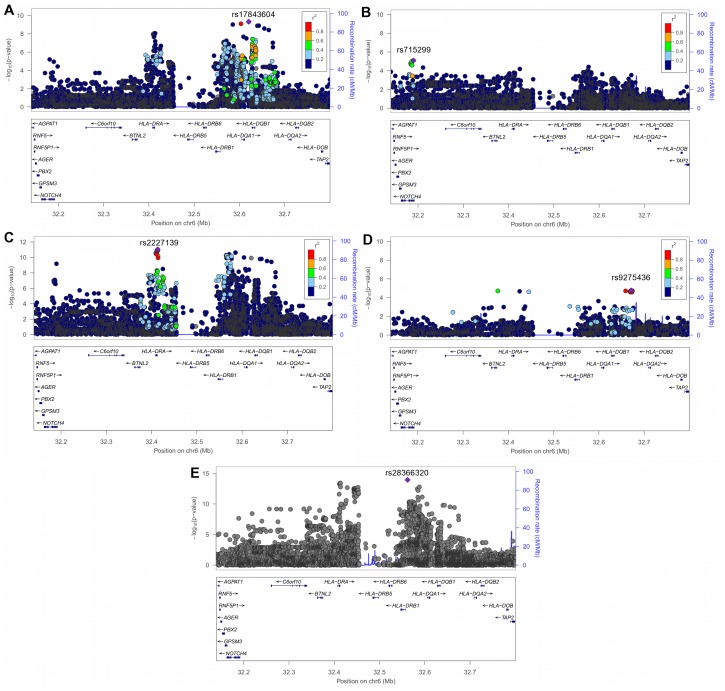
Regional association plots of SNP-sarcoidosis association test results within the MHC Class II region. (A–D) Association results in the AA discovery set (A), AA replication set (B), a meta-analysis between the AA discovery and AA replication sets (C), the EA dataset (D), and a meta-analysis of the AA discovery, AA replication and EA datasets (E). Each SNP is colored according to its LD (*r* ^2^) with the top SNP, except for (E) since the meta-analysis was performed on two different populations. The recombination rate is denoted by the blue solid line. Plots were drawn using LocusZoom [100].

A total of 864,829 single-nucleotide polymorphisms (SNPs) in our AA discovery set passed quality control assessment (Materials and Methods, Figure 1, Table 1). To increase the density of SNPs to be tested for association, we performed genotype imputation across the genome with the 1000 Genomes Project Phase I haplotypes as reference (Materials and Methods). The GWAS of the AA discovery set demonstrated no evidence for inflation of the test statistics (genomic control inflation factor [λ_GC_] = 0.980) after comparing the observed and expected distributions of the SNP-sarcoidosis association *P*-values calculated using EMMAX (Figure S1, Materials and Methods). This suggests our regression model was able to account for population stratification in this dataset. The quantile-quantile plot revealed the presence of significant genetic effects associated with sarcoidosis (Figure S1). This dataset had good statistical power (at α = 5×10^−8^) to detect associations from common alleles with odds ratios ≥1.5 (Figure S2). We only found variants within previously reported MHC Class II genes [11], [22] exceeding genome-wide significance in this dataset (Figure 2A, Figure 3A, Table S2); *HLA-DRA* with the peak signals at multiple SNPs in perfect linkage disequilibrium (LD) with each other (*r* ^2^ = 1) including a missense SNP rs7192 (*P*_AA-Disc_ = 8.73×10^−9^), *HLA-DQA1* (peak signal at rs17843604, *P*_AA-Disc_ = 4.77×10^−10^), and *HLA-DQB1* (peak signal at rs149288329, *P*_AA-Disc_ = 1.27×10^−9^) (Table S2). These SNPs were not LD with each other (*r* ^2^≤0.054).

**Table 1:** Sample summary before and after quality control (QC).

	African American				European American	
Characteristic	All samplesbefore QC	Discovery setafter QC	Replication setafter QC	All Samples after QC	Replication set before QC	Replication set after QC
Cases	1487	818	455	1273	518	442
Controls	1504	908	577	1465	379	339
External Controls	180^a^	180	0	180	3208^b^	1945
Unknown Affection Status	2	0	0	0	0	0
Male	889	575	244	819	1847	1173
Female	2264	1331	768	2099	2247	1553
Unknown Gender	20	0	0	0	11	0
Total	3173	1906	1012	2918	4105	2726

a Taken from the Illumina YRI-ASW iControlDB;

b 175 Caucasian healthy controls from the Illumina iControlDB, 1047 controls from the dbGaP GENEVA Melanoma study, and 1986 controls from the dbGAP CIDR: NGRC Parkinson’s Disease Study.

### Genome-wide Meta-Analysis of the AA Discovery and Replication Sets

After assessing association between SNPs and sarcoidosis using logistic regression in the AA replication set (Materials and Methods, Figure 1, Table 1), we found little evidence for inflation of the test statistics in this dataset (λ_GC_ = 1.030, Figure S1). A meta-analysis of the AA discovery and replication sets yielded additional MHC SNPs that surpassed genome-wide significance in the meta-analysis results not present in either set alone. These included a genotyped SNP in the previously unreported neurogenic locus notch homolog protein 4 (*NOTCH4*) gene (rs715299, *P*_AA-meta_ = 6.51×10^−10^) and other SNPs within the MHC Class II genes (Figure 1B, Figure 3C, Table 2, Table S2).

**Table 2:** Regions of association meeting genome-wide significance and their most significant SNPs grouped by sample.

CHR	BP(hg 19)	SNP	Gene	Alleles^1^	African Americans							European Americans			P_All-Meta_	Heterogeneity Test	
					MAF_AA-Disc_ 2	OR_AA-Disc_ 3	*P*_AA-Disc_	MAF_AA-Rep_ 2	OR_AA-Rep_ 3	*P*_AA-Rep_	P_AA-Meta_	MAF_EA_ 2	OR_EA_ 3	*P*_EA_		*Q*	*I* 2 (%)
6	32,411,646	rs7192	*HLA-DRA* a	G/T	0.424	1.66	8.73E-09	0.445	1.40	3.44E-04	1.40E-11	0.395	1.35	1.26E-04	5.28E-14	0.304	16
6	32,620,283	rs17843604	*HLA-DQA1* a	C/T	0.402	0.63	4.77E-10	0.378	0.80	1.70E-02	1.21E-10	0.56	0.91	1.81E-01	2.73E-08	5.33E-05	89.8
6	32,642,794	rs149288329	*HLA-DQB1* a	T/C	0.025	1.92	1.27E-09	0.038	1.87	1.15E-02	1.55E-10	NA	NA	NA	NA	NA	NA
6	32,189,841	rs715299	*NOTCH4* b	T/G	0.454	1.30	1.12E-05	0.480	1.52	8.14E-06	6.51E-10	0.324	1.14	9.58E-02	2.15E-08	0.064	63.6
6	31,272,612	rs6457375	*HLA-C* c	A/G	0.423	0.88	4.24E-01	0.403	1.17	9.06E-02	7.26E-01	0.49	1.58	1.98E-09	9.80E-06	1.84E-05	90.8
6	31,326,324	rs2596475	*HLA-B* c	T/C	0.287	0.90	5.27E-01	0.263	1.00	9.84E-01	6.01E-01	0.386	1.52	3.82E-08	2.72E-05	7.45E-05	89.5
6	32,446,853	rs17203612	*HLA-DRB5* c	T/C	0.270	0.64	2.66E-05	0.243	0.79	2.42E-02	2.33E-06	0.438	0.63	1.82E-08	2.80E-13	0.209	36.1

1 Major/minor allele of AAs as the reference;

2 Minor allele frequency;

3 The odds ratio (OR) was calculated with respect to the minor allele of AAs.

a Previously reported sarcoidosis loci meeting genome-wide significance in the AA discovery set.

b Potentially novel region meeting genome-wide significance after the meta-analysis of AA datasets.

c Previously reported sarcoidosis loci meeting genome-wide significance in the EA dataset.

Note that stepwise conditional analysis results to identify independent signals within the MHC region can be found in Tables S3 and S4.

### Stepwise Conditional Association of the MHC Region in Combined AA Dataset

Since the MHC region is known for its extensive regions of high LD [29], we sought to assess whether the novel AA association signal within *NOTCH4* was independent of the signals within the MHC Class II genes. We performed stepwise conditional association analyses (Materials and Methods) among variants with *P*_AA-meta_ <5×10^−8^ in the MHC region in the combined AA set and at step one used the most significant SNP (rs2227139, *HLA-DRA*) as the covariate. After adjusting for this *HLA-DRA* SNP, we observed significant residual associations in several other regions; the most significant of which was at rs146146117 (*HLA-DQA1*, *P*_conditional_ = 6.81×10^−8^, Table S3). Significant residual associations remained after the next step of adjusting for *HLA-DRA* and *HLA-DQA1* SNPs; the most significant residual association was within *HLA-DRB1* (rs9461776, *P*_conditional_ = 1.45×10^−7^, Table S3). We continued to step three by adding this *HLA-DRB1* SNP into the regression and found the most significant residual signals at *NOTCH4* (rs715299, *P*_conditional_ = 1.74×10^−6^) and *HLA-DQA1* (rs9272320, *P*_conditional_ = 7.04×10^−6^) (Table S3). The subsequent (and final) step adding this *HLA-DQA1* SNP (rs9272320) as a covariate resulted in diminished association signals for the remaining significant SNPs within the MHC class II genes (*P*_conditional_ ≥0.014), whereas *NOTCH4* remained significant (rs715299, *P*_conditional_ = 8.85×10^−5^) (Table S3). While the *P*-value for *NOTCH4* did not retain the GWAS threshold of 5×10^−8^ after rigorous conditioning, it remains the only significant effect well exceeding the suggestive level of association. It suggests that the observed signal within *NOTCH4* is independent of the evaluated SNPs within the MHC Class II genes. These analyses also showed the existence of multiple independent signals within this MHC region (Table 2).

### Confirmation of Previously Reported SNPs Associated with Sarcoidosis in the Combined AA Datasets

Three significant SNPs reported in the previous German GWAS in the MHC region (*P*<1×10^−6^) [11] were also replicated in our combined AA datasets (rs7194 [in perfect LD with rs7192], *HLA-DRA*, *P*_AA-meta_ = 1.40×10^−11^; rs9268853, *HLA-DRB5*, *P*_AA-meta_ = 7.40×10^−4^; and rs615672, *HLA-DRB1*, *P*_AA-meta_ = 2.60×10^−9^, Table 3). The previously reported peak SNP within *BTNL2* (rs2076530) [9], [11], [25] was not strongly associated with sarcoidosis in our AA datasets (*P*_AA-meta_ = 0.024, Table 3). However, a SNP with 4 kb upstream of rs2076530, rs9268482, was suggestive of association (*P*_AA-meta_ = 6.32×10^−6^, Table 3). Interestingly, we also identified a suggestive association at a *BTNL2* coding-synonymous SNP, rs9268480 (*P*_AA-meta_ = 1.03×10^−5^), only 28 bp upstream of rs2076530 and in high LD with rs9268482 (*r* ^2^ = 0.996). Since *BNTL2* is only 170 kb apart from *NOTCH4*, we sought to assess whether the signal within *NOTCH4* is independent of the signal within *BTNL2* using conditional association analyses. When adjusting for one of those associated *BTNL2* SNPs (rs9268482), we found *NOTCH4* remained significant (rs715299, *P*_conditional_ = 2.86×10^−8^). On the other hand, after adjusting for the *NOTCH4* SNP, we still observed a significant residual signal at the *BTNL2* SNP (rs9268482, *P*_conditional_ = 1.26×10^−4^). These indicated the signal within *NOTCH4* is also independent of the *BTNL2* signal.

**Table 3:** Replication of previously reported SNPs associated with sarcoidosis [9], [11], [25], [27].

CHR	BP(hg 19)	SNP	Gene	Alleles^1^	African Americans							European Americans			P_All-Meta_	Heterogeneity Test	
					MAF_AA-Disc_ 2	OR_AA-Disc_	*P*_AA-Disc_	MAF_AA-Rep_ 2	OR_AA-Rep_	*P*_AA-Rep_	P_AA-Meta_	MAF_EA_ 2	OR_EA_	*P*_EA_		*Q*	*I* 2 (%)
6	32,363,816	rs2076530	*BTNL2*	T/C	0.309	0.84	2.50E-01	0.312	0.80	2.46E-02	2.42E-02	0.434	0.70	4.19E-06	1.44E-06	0.324	11.3
6	32,412,480	rs7194	*HLA-DRA*	A/G	0.424	1.66	8.73E-09	0.445	1.40	3.44E-04	1.40E-11	0.395	1.35	1.26E-04	5.28E-14	0.304	16
6	32,429,643	rs9268853	*HLA-DRB5*	T/C	0.214	0.72	1.16E-03	0.197	0.86	2.03E-01	7.40E-04	0.331	0.76	9.79E-04	2.39E-06	0.544	0
6	32,574,171	rs615672	*HLA-DRB1*	C/G	0.449	0.64	1.23E-06	0.438	0.72	5.50E-04	2.60E-09	0.643	0.81	8.00E-03	9.97E-10	2.00E-07	93.5
6	57055354	rs1040461	*RAB23*	C/T	0.158	1.13	3.24E-02	0.177	1.21	1.18E-01	8.04E-03	0.079	0.89	4.18E-01	1.80E-01	0.257	26.4
10	81,926,702	rs1049550	*ANXA11*	G/A	0.185	0.68	7.91E-04	0.187	0.88	2.89E-01	8.46E-04	0.409	0.81	8.33E-03	2.30E-05	0.356	3.2

1 Major/minor allele of AAs as the reference;

2 Minor allele frequency;

3 The odds ratio (OR) was calculated with respect to the minor allele of AAs.

We saw modest association with two other previously reported susceptibility genes: *ANXA11* [11] and *RAB23* [27]. A non-synonymous SNP within *ANXA11*, rs1049550, was associated with sarcoidosis in our combined AA datasets at *P*_AA-meta_ = 8.46×10^−4^ (Table 3). A similar modest association was seen with a non-synonymous SNP within *RAB23* (rs1040461, *P*_AA-meta_ = 8.04×10^−3^, Table 3). We did find suggestive evidence of association on 5q11.2 (peak signal at rs116137605 within a region between *SNX18* and *ESM1*, *P*_AA-meta_ = 3.09×10^−5^) a region identified in our previous linkage and fine-mapping studies [10], [28], [30].

### Genome-wide Association Scan of EA Dataset

We found 682,921 genotyped SNPs passed quality control measures in our EA dataset (Materials and Methods, Figure 1, Table 1). After performing imputation with the 1000 Genomes Project haplotypes, the SNP-sarcoidosis association calculated using logistic regression of the EA dataset showed little evidence for inflation of the test statistics (λ_GC_ = 1.027, Figure S1). This dataset also had good statistical power (at α = 5×10^−8^) to detect associations from common alleles with odds ratios ≥1.5 (Figure S2). We observed genome-wide significance SNPs within previously reported MHC genes [9], [11], [24] including *HLA-C* (peak signal at rs6457375, *P*_EA_ = 1.98×10^−9^), *HLA-B* (peak signal at rs2596475, *P*_EA_ = 3.82×10^−8^), and *HLA-DRB5* (peak signal at rs17203612, *P*_EA_ = 1.82×10^−8^) (Figure 2C, Figure 3D, Table 2, Table S2). However, we did not find any variant within *NOTCH4* passed genome-wide significance in this dataset (Figure S3). Stepwise conditional association analyses further demonstrated two independent signals exist within this region tagged by rs6457375 (*HLA-C*) and rs17203612 (*HLA-DRB5*) (Table S4).

### Confirmation of Previously Identified Loci in EA Dataset

We replicated significant SNPs from the German GWAS [11] in the EA dataset including rs7194 (*HLA-DRA*, *P*_EA_ = 1.26×10^−4^), rs9268853 (*HLA-DRB5*, *P*_EA_ = 9.79×10^−4^), rs615672 (*HLA-DRB1*, *P*_EA_ = 8.00×10^−3^), and rs1049550 (*ANXA11*, *P*_EA_ = 8.33×10^−3^) (Table 3). We also replicated the *BTNL2* SNP, rs2076530 [9], [11], [25], in our EA dataset (*P*_EA_ = 4.19×10^−6^, Table 3). We did not, however, confirm the *RAB23* association [27] in this dataset (rs1040461, *P*_EA_ = 0.418, Table 3).

### Meta-analysis Results of All Datasets

Among regions that met genome-wide significance in the AA meta-analysis, we also found significant associations within *HLA-DRA*, *HLA-DRB1*, and *HLA-DQA1* in the EA dataset (8.25×10^−5^≤*P*_EA_ ≤3.97×10^−2^, 3.77×10^−14^≤*P*_All-meta_ ≤7.23×10^−8^) (Figure 3E, Table S2). We found a weak association to the *NOTCH4* SNP (rs715299) in the EA dataset (*P*_EA_ = 0.096), perhaps suggesting its ethnicity specific effect (the Cochran’s *Q* test of heterogeneity *P* = 0.064 and the inconsistency index *I* ^2^ = 63.60%, see Materials and Methods). Conversely, when evaluating regions reaching genome-wide significant in the EA dataset, variants within *HLA-DRB5*, *HLA-DRB1,* and *HLA-DQA1* were also significant in the AA datasets (1.81×10^−7^≤*P*_AA-meta_ ≤1.28×10^−5^, 1.16×10^−14^≤*P*_All-meta_ ≤2.65×10^−12^, Table S2), whereas *HLA-C* and *HLA-B* were not (*P*_AA-meta_ ≥0.575, Table S2).

### Suggestive Association Regions

We observed multiple regions reached suggestive association (*P*_all-meta_ <1×10^−4^) in the meta-analysis of all AA and EA datasets. These included variants within *TRAK1, SLC44A4*, *GLI3*-*C7orf25*, *ATP8A2*, and *TGM3* (Tables S5). We observed additional suggestive association regions (*P*<1×10^−4^) that were unique to one ethnic group. For example, we identified variants with suggestive association within *FHIT*, *PRDM1*, *FRMD3*, *DMBT1* and a region between *ZSCAN2* and *ALPK3* in the combined AA datasets only (Tables S5). We also observed suggestive association only in the EA dataset within *CASP10*, *RARB,* and *NCR3* among others (Tables S5). Several of these suggestive effects fall within genes implicated in other lung or inflammatory diseases (Table S6).

## Discussion

Previously reported GWASs of sarcoidosis have been limited to European (specifically German) samples. Ours is the first GWAS of sarcoidosis in Americans and, even more importantly, of AAs, the population most commonly and severely affected. Our results, while demonstrating some shared effects across ethnicities, strongly support the presence of ethnic specific genetic effects. We identified significant association between sarcoidosis and a previously unreported locus (*NOTCH4*) in our AA datasets. This association was determined to be independent of other neighboring MHC genes and is an attractive biological candidate. *NOTCH4* encodes a member of the Notch family that is involved in controlling cell fate decisions during developmental processes and regulating the activity of T cell immune responses [31], [32]. The Notch signaling pathway also plays a role in endothelial cell differentiation, apoptosis and proliferation [33], [34], [35], [36]. Further, *NOTCH4* is highly expressed in the lung and may play a key role in the lung development and diseases such as asthma and lung arteriovenous shunts [37], [38], [39], [40], [41]. *NOTCH4* has also been associated with neonatal lupus [42], multiple sclerosis [43], systemic sclerosis [44], and other immune-related disorders [45], [46], [47], [48]. We also saw evidence of suggestive association of *NOTCH4* in our EA dataset. While further studies are needed to define the role of *NOTCH4* in the specific pathogenesis of sarcoidosis, a novel association to this gene is supported by previous expression and disease studies.

We replicated associations for several previously reported sarcoidosis susceptibility risk loci in our AA collection including MHC Class II region genes (*HLA-DRA*, *HLA-DRB5*, *HLA-DRB1,* and *HLA-DQA1*), *BTNL2*, *RAB23,* and *ANXA11* [9], [11], [25], [27], [49]. These regions were also replicated in our EA dataset except for *RAB23*. It is known that the MHC Class II region plays a major role in immune-mediated disorders, including associations to celiac disease, insulin-dependent diabetes mellitus, rheumatoid arthritis, multiple sclerosis, and systemic lupus erythematosus (SLE) [50], [51]. Similarly, *BTNL2*, *RAB23,* and *ANXA11* have been suggested to play a role in T-cell activation [9], antibacterial defense processes [27], and apoptosis [11]. It is worth noting that we did not replicate the association with *C10orf67* [12] as identified in a joint GWAS of German patients with either sarcoidosis or Crohn’s disease.

Additional regions with suggestive evidence of association in both AAs and EAs include *TRAK1, SLC44A4*, *GLI3*-*C7orf25*, *ATP8A2*, and *TGM3*. While the biological relevance of most of these genes to sarcoidosis is still unknown, *GLI3*-*C7orf25* and *TGM3* may warrant further investigation. Although *C7orf25* is a hypothetical gene with unknown function, *GLI3* encodes zinc finger protein Gli3 that has a bipotential function as a transcriptional activator or repressor of the sonic hedgehog pathway [52], [53]. This pathway contains *RAB23* (discussed above) and has been suggested to play a role in the sarcoidosis pathophysiology [27]. *TGM3* (Transglutaminase 3) encodes protein involved in the later stages of cell envelope formation in the epidermis and hair follicle [54] and has been associated with celiac disease [55], [56] and psoriasis [57], [58].

Despite the overlap of compelling signals across populations, we did find evidence of genetic heterogeneity between ethnic groups in this disease (see Tables 2 and 3). The previously reported MHC Class I region [24] including *HLA-C* and *HLA-B* (associated with psoriasis [59] and ankylosing spondylitis [60], respectively) was associated only in the EA dataset. Other noteworthy genes with suggestive association specific to EAs included *CASP10*, *RARB,* and *NCR3*. *CASP10 (*caspase 10) plays a role in apoptosis and has been associated with autoimmune lymphoproliferative syndrome [61] and non-Hodgkin lymphoma [62]. In addition, *RARB* (retinoic acid receptor beta) and *NCR3* (natural cytotoxicity triggering receptor 3) have been associated with pulmonary function based on a recent GWAS of European Caucasians [63]. Suggestive associations specific to AAs include *FHIT*, *FRMD3, DMBT1, and PRDM1*. *FHIT* (fragile histidine triad) is involved in various intracellular functions and a putative tumor suppressor for various cancers including lung cancer [64], [65]. *FRMD3* (FERM domain containing 3) is over-expressed in normal human lung tissue compared with tissue from lung tumors of lung carcinoma patients suggesting its important role in the origin and progression of lung cancer [66]. *DMBT1* (deleted in malignant brain tumors 1) is overexpressed in epithelial cells [67] and has been found associated with ulcerative colitis [68] and Crohn’s disease [67], [69]. *PRDM1* (PR domain containing protein 1) plays a role as a repressor of beta-interferon gene expression [70] and had been associated with rheumatoid arthritis [71], inflammatory bowel disease (IBD) [72], [73], and SLE [74], [75]. We also observed variants with suggestive associations specific to AAs in a region containing *ZSCAN2*, *SCAND2*, *WDR73*, *NMB*, *SEC11A*, *ZNF592*, and *ALPK3* as well as a region identified in our linkage studies [10], [28], [30] on 5q11.2 (a region between *SNX18* and *ESM1*). However, the actual biological functions of these genes are largely unknown.

In summary, this is the first report of GWAS in an American sample and the first report of a significant association between sarcoidosis and *NOTCH4*. We have replicated several previously reported sarcoidosis susceptibility loci in both our EA and AA samples as well as report several biologically plausible effects at loci with suggestive statistical evidence. We report sarcoidosis associations both shared between ethnicities as well as those unique to either our AA or EA dataset, supporting genetic heterogeneity of this disease. The presence of genetic heterogeneity may well serve as a useful tool in the isolation of the causal variants associated with this disease as it has in other complex disorders [76], [77]. Finally, this study demonstrates both the usefulness of and need for genetic studies of sarcoidosis in diverse populations and further elucidates potential pathogenic mechanisms of this disease. Future replication, sequencing and functional studies are required to further elucidate the causal variants that may underlie these associations as well as to discover rare variants that may have yet to be identified.

## Materials and Methods

### Ethics Statement

The study and sample collection were approved by the Institutional Review Board (IRB) at all participating institutions including A Case Control Etiologic Study of Sarcoidosis (ACCESS) Group, Sarcoidosis Genetic Analysis (SAGA) study, Henry Ford Health System in Detroit, Michigan, and Oklahoma Medical Research Foundation (OMRF), Oklahoma City, Oklahoma, Institutional Review Boards (IRBs). Only individuals who signed informed consent forms were included in this study. No minors or children were involved in our study.

### Subjects

Our AA sample collection, which comprises 1487 cases and 1504 controls (Figure1, Table 1), was taken from an extensive cohort of AA sarcoidosis patients, family members and controls assembled from 1) case-control pairs collected as a part of a 10 center collaborative study (ACCESS Group) [78], 2) the SAGA sample ascertained through affected sib pairs [79], 3) a nuclear family-based sample ascertained through single sarcoidosis-affected offspring from the Henry Ford Health System in Detroit, Michigan [80], and 4) healthy controls from the OMRF Lupus Family Registry and Repository (LFRR) [81]. The AA cases and their family members were grouped into a discovery set of 818 cases and 908 related and unrelated controls and the other 455 independent cases and 557 independent controls were selected for a replication set after applying quality control measures as described below (Figure 1, Table 1). In addition, genotype data from 180 HapMap controls from Yoruba in Ibadan, Nigeria (YRI) and of African ancestry in Southwest USA (ASW) were obtained from the Illumina HumanOmni1-Quad iControlDB (http://www.illumina.com/science/icontroldb.ilmn) and included into the control group of the AA discovery set, as is common practice in order to increase statistical power [82], [83], [84]. The EA dataset consisted of 518 independent cases and 379 independent controls from the ACCESS and the Henry Ford Health System studies mentioned above. We also assembled external genotype data on 3208 healthy Caucasian controls from the Illumina iControlDB (175), the dbGaP (Accession: phs000187.v1.p1) GENEVA Melanoma study (1047), and the dbGAP (Accession: phs000196.v2.p1) CIDR: NGRC Parkinson’s Disease Study (1986) (Figure 1, Table 1). Each sample collection site received the IRB approval to recruit samples. All samples were processed and genotyped at the OMRF under the auspice of the OMRF IRB.

### Genotyping and Quality Control

Genotyping was performed at the OMRF using the Illumina HumanOmni1-Quad array for ∼1.1M variants across the genome. SNPs had to meet the following quality control criteria for inclusion for each population: well-defined cluster plots by visual inspections, call rate >95%, minor allele frequency >0.01, Hardy-Weinberg proportion tests *P*>0.0001 in cases and *P*>0.001 in controls, and case-control differences in missingness *P*>0.001. Copy number variations, X, Y, XY, and mitochondrial chromosomes were not included in the analysis. A total of 864,829 and 682,921 SNPs passed our quality controls in the AA discovery and replication sets and the EA dataset, respectively. We found 657,350 successfully genotyped SNPs that overlap between the panels. Samples were removed from analysis if they were determined to be a duplicate of another sample, cryptic relatedness in the independent datasets (the proportion of alleles shared identical by descent >0.25), displayed low call rates (<90%), exhibited extreme heterozygosity (>5 standard deviations from the mean), demonstrated either outlying principal component values of population membership calculated by EIGENSOFT 3.0 [85] or global ancestry estimates calculated by ADMIXMAP [86], [87], or revealed discrepancies between reported gender and genetic data (Table S1). For the EA dataset, we assigned to each sarcoidosis case the five best-matched controls as determined by identity-by-state (IBS) allele sharing using PLINK v1.07 [88] resulting in a large drop-out of external controls in the EA dataset.

### Imputation Method

Imputation was performed in each population at 5 Mb bins across the genome using the IMPUTE2 program [89], [90]. The 1000 Genomes Project Phase I data release (June 2011), which contains haplotypes derived from 1,094 individuals from Africa, Asia, Europe, and the Americas, was used as the reference [89], [90]. IMPUTE2 estimated the posterior probabilities for the three possible genotypes (i.e. AA, AB, and BB). The posterior probabilities were then converted to the most likely genotypes with a threshold of 0.9. Imputed SNPs with either low imputation accuracy (information measure <0.5 and the average maximum posterior genotype call probability <0.9) and that failed the SNP quality control standards described above were removed in order to minimize false positives. After imputation, 10,948,298 SNPs in the AA discovery set, 11,160,451 SNPs in the AA replication set, and 6,620,482 SNPs in the EA replication set passed quality control measures for analysis.

### Association Analyses

Because our discovery set contained related individuals, association analysis to any single marker in this set was performed using the Efficient Mixed-Model Association eXpedited (EMMAX) software [91], [92]. EMMAX was chosen because it implements a variance component approach in the linear mixed-model that simultaneously adjusts for both pairwise genetic relatedness between individuals and corrects for population stratification using an empirical kinship matrix based on the proportion of alleles at all genome-wide SNPs shared identical-by-state between all pairs of individuals in the study [91]. We assumed an additive model [91], [92] and adjusted the statistics for gender. Since EMMAX does not calculate odds ratios (ORs), we estimated these using logistic regression as implemented in PLINK using independent samples (480 cases and 367 controls) ascertained from the AA discovery set. The association analyses of the independent sets of AAs and EAs were calculated using logistic regression in PLINK. We assumed the additive genetic model and adjusted the statistics for gender and the first five principal components of each population (calculated using EIGENSOFT 3.0). Meta-analyses were performed using the weighted *Z-*score method that accounts for the direction of effects and sample-size as implemented in METAL [93]. Both the Cochran’s *Q* test statistic and *I* ^2^ index were used to test for heterogeneity in the meta-analysis of all samples. The Cochran’s *Q* test calculates the weighted sum of the squared deviations between each study effects and the overall effect across studies [94], whereas the *I* ^2^ index quantifies the percentage of inconsistency across studies due to heterogeneity rather than by chance [95]. The *Q* test with *P*<0.05 or *I* ^2^>50% indicates the presence of heterogeneity. Stepwise conditional association analysis in AAs was conducted for SNPs with *P*<5×10^−8^ using EMMAX adjusting for gender and SNPs of interest, a SNP added at a time. We required a SNP threshold of *P*<5×10^−8^ to be considered significantly associated and *P*<1×10^−4^ to be considered suggestively associated with sarcoidosis [96], [97], [98].

The power calculations for different minor allele frequencies and odds ratios for each dataset were performed using the Genetic Power Calculator program [99] and have been summarized in Figure S2. The assumptions are a disease prevalence of 0.05%, complete linkage disequilibrium between SNP and predisposing loci, an additive genetic model and a type I error rate α = 5×10^−8^. To present power curves that are comparable across sets, we used a power calculator that assumes independence, but adjusted the analysis of the AA discovery set (family-based set) assuming a familial correlation of 0.25 since most pairs are siblings (and thus smaller equivalent count or 75% of the total cases and controls in this set).

## Supporting Information

Figure S1 The quantile-quantile (Q–Q) plots of the observed and expected distributions of *P*-values. (A–C) The Q–Q plots for (A) the AA discovery set (genomic control inflation factor [λ_GC_]  = 0.980), (B) the AA replication set (λ_GC_ = 1.030), and (C) the EA dataset (λ_GC_ = 1.027).
(DOC)

Figure S2 Power calculation plots of the GWAS datasets. (A–C) Power calculation plots for the AA discovery set (A), the AA replication set (B), and the EA dataset (C).
(DOC)

Figure S3 Regional association plots of SNP-sarcoidosis association test results within *NOTCH4*. (A–D) Association results in the AA discovery set (A), AA replication set (B), a meta-analysis between the AA discovery and AA replication sets including the LD (*D*’) plot (C), and the EA dataset including the LD (*D*’) plot (D). Each SNP is colored according to its LD (*r* ^2^) with the top SNP. The blue solid line denotes the recombination rate.
(DOC)

Table S1 Summary of dropped samples after QC.
(DOC)

Table S2 Association results with *P*<5×10^−8^ in either dataset.
(XLS)

Table S3 Stepwise conditional analysis in AA samples for SNPs in the MHC region with *P*<5×10^−8^.
(XLS)

Table S4 Stepwise conditional analysis in EA samples for SNPs in the MHC region with *P*<5×10^−8^.
(XLS)

Table S5 Association results with *P*<1×10^−4^ in either dataset.
(XLS)

Table S6 Shared or Ethnic Specific Suggestive Association Regions supported by the heterogeneity test results and list of inflammatory or lung diseases associated with these regions.
(DOC)
