# Correction: Genome-Wide Association Study of African and European Americans Implicates Multiple Shared and Ethnic Specific Loci in Sarcoidosis Susceptibility

**DOI:** 10.1371/annotation/800aa394-fb39-471b-b5c5-b648079921a4

**Published:** 2013-09-10

**Authors:** Indra Adrianto, Chee Paul Lin, Jessica J. Hale, Albert M. Levin, Indrani Datta, Ryan Parker, Adam Adler, Jennifer A. Kelly, Kenneth M. Kaufman, Christopher J. Lessard, Kathy L. Moser, Robert P. Kimberly, John B. Harley, Michael C. Iannuzzi, Benjamin A. Rybicki, Courtney G. Montgomery

The authors wish to add the following to the Funding Statement:

"The dbGAP CIDR:NGRC Parkinson’s disease study dataset(s) (Accession: phs000196.v2.p1):

This work utilized in part data from the NINDS DbGaP database from the CIDR:NGRC Parkinson’s Disease Study. Funding support for this study was provided through the the National Institute of Neurological Disorders and Stroke, National Institutes of Health (5R01NS036960-10). Funding source for genotyping was provided through the National Institutes of Health (HHSN268200782096C).

The dbGaP High Density SNP Association Analysis of Melanoma: Case-Control and Outcomes Investigation dataset(s) (Accession: phs000187.v1.p1):

This work utilized in part data from the DbGaP database from the High Density SNP Association Analysis of Melanoma: Case-Control and Outcomes Investigation study. Research support to collect data and develop an application to support this project was provided by the National Cancer Institute (3P50CA093459, 5P50CA097007, and 5R01CA133996) and the National Institute of Environmental Health Sciences, National Institute of Health (5R01ES011740). Funding source for genotyping was provided through the National Institutes of Health (HHSN268200782096C)."

