# Supplementary material for: Genome-Wide Association Study of African and European Americans Implicates Multiple Shared and Ethnic Specific Loci in Sarcoidosis Susceptibility
Source: PLoS One. 2012 Aug 27;7(8):e43907. doi: 10.1371/journal.pone.0043907 (PMC3428296; doi:10.1371/journal.pone.0043907)
Supplement: Figure S1 — The quantile-quantile (Q–Q) plots of the observed and expected distributions of P-values. (A–C) The Q–Q plots for (A) the AA discovery set (genomic control inflation factor [λGC] = 0.980), (B) the AA replication set (λGC = 1.030), and (C) the EA dataset (λGC = 1.027). (DOC) [file pone.0043907.s001.doc]

**Figure S1**. **The quantile-quantile (Q-Q) plots of the observed and expected distributions of *P*-values.**

(A-C) The Q-Q plots for (A) the AA discovery set (genomic control inflation factor [λ_GC_] = 0.980), (B) the AA replication set (λ_GC_ = 1.030), and (C) the EA dataset (λ_GC_ = 1.027).
