# Supplementary material for: Genome-Wide Association Study of African and European Americans Implicates Multiple Shared and Ethnic Specific Loci in Sarcoidosis Susceptibility
Source: PLoS One. 2012 Aug 27;7(8):e43907. doi: 10.1371/journal.pone.0043907 (PMC3428296; doi:10.1371/journal.pone.0043907)
Supplement: Figure S2 — Power calculation plots of the GWAS datasets. (A–C) Power calculation plots for the AA discovery set (A), the AA replication set (B), and the EA dataset (C). (DOC) [file pone.0043907.s002.doc]

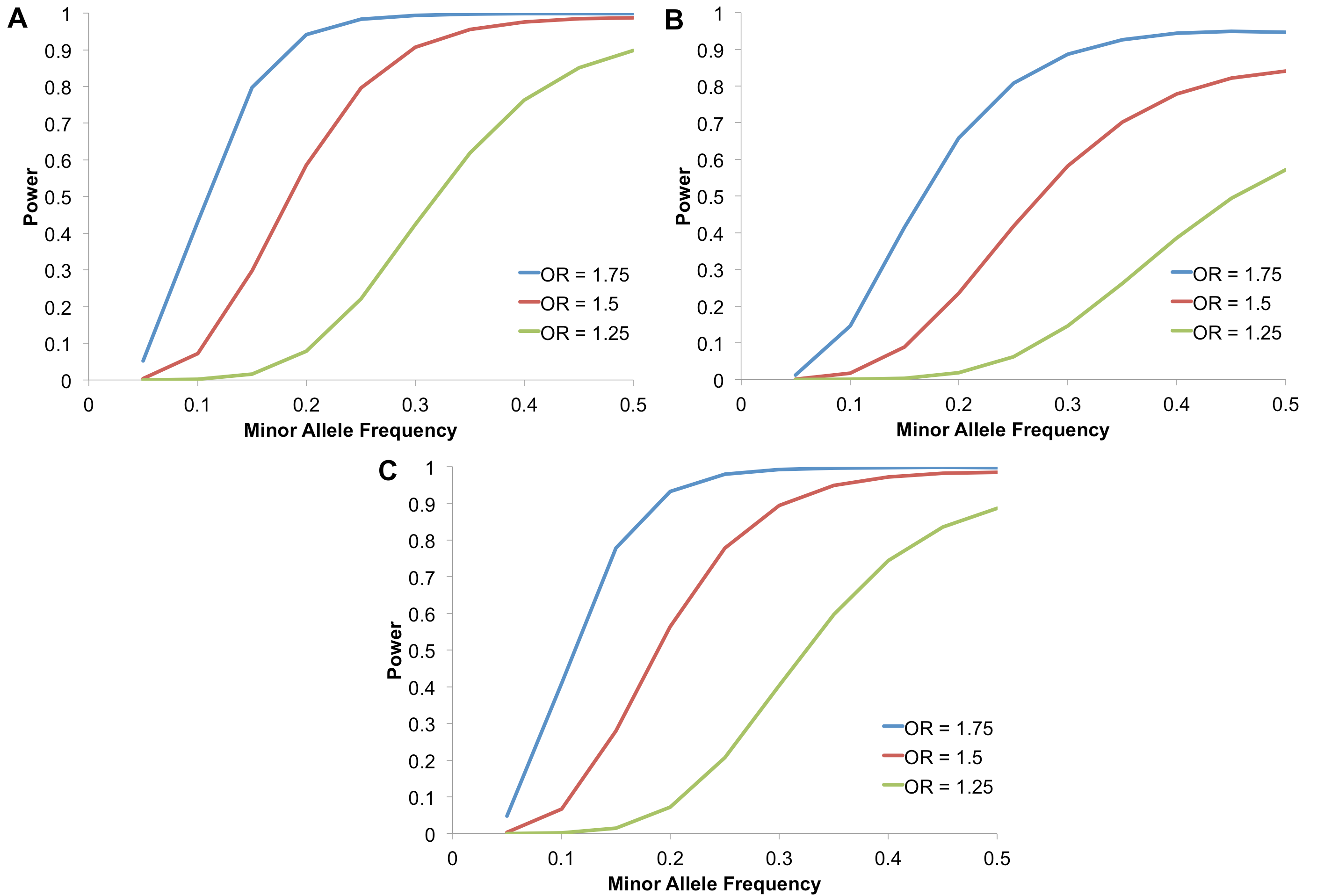


**Figure S2. Power calculation plots of the GWAS datasets.**

(A-C) Power calculation plots for the AA discovery set (A), the AA replication set (B), and the EA dataset (C).
