# Supplementary material for: Genome-Wide Association Study of African and European Americans Implicates Multiple Shared and Ethnic Specific Loci in Sarcoidosis Susceptibility
Source: PLoS One. 2012 Aug 27;7(8):e43907. doi: 10.1371/journal.pone.0043907 (PMC3428296; doi:10.1371/journal.pone.0043907)
Supplement: Figure S3 — Regional association plots of SNP-sarcoidosis association test results within NOTCH4. (A–D) Association results in the AA discovery set (A), AA replication set (B), a meta-analysis between the AA discovery and AA replication sets including the LD (D’) plot (C), and the EA dataset including the LD (D’) plot (D). Each SNP is colored according to its LD (r 2) with the top SNP. The blue solid line denotes the recombination rate. (DOC) [file pone.0043907.s003.doc]

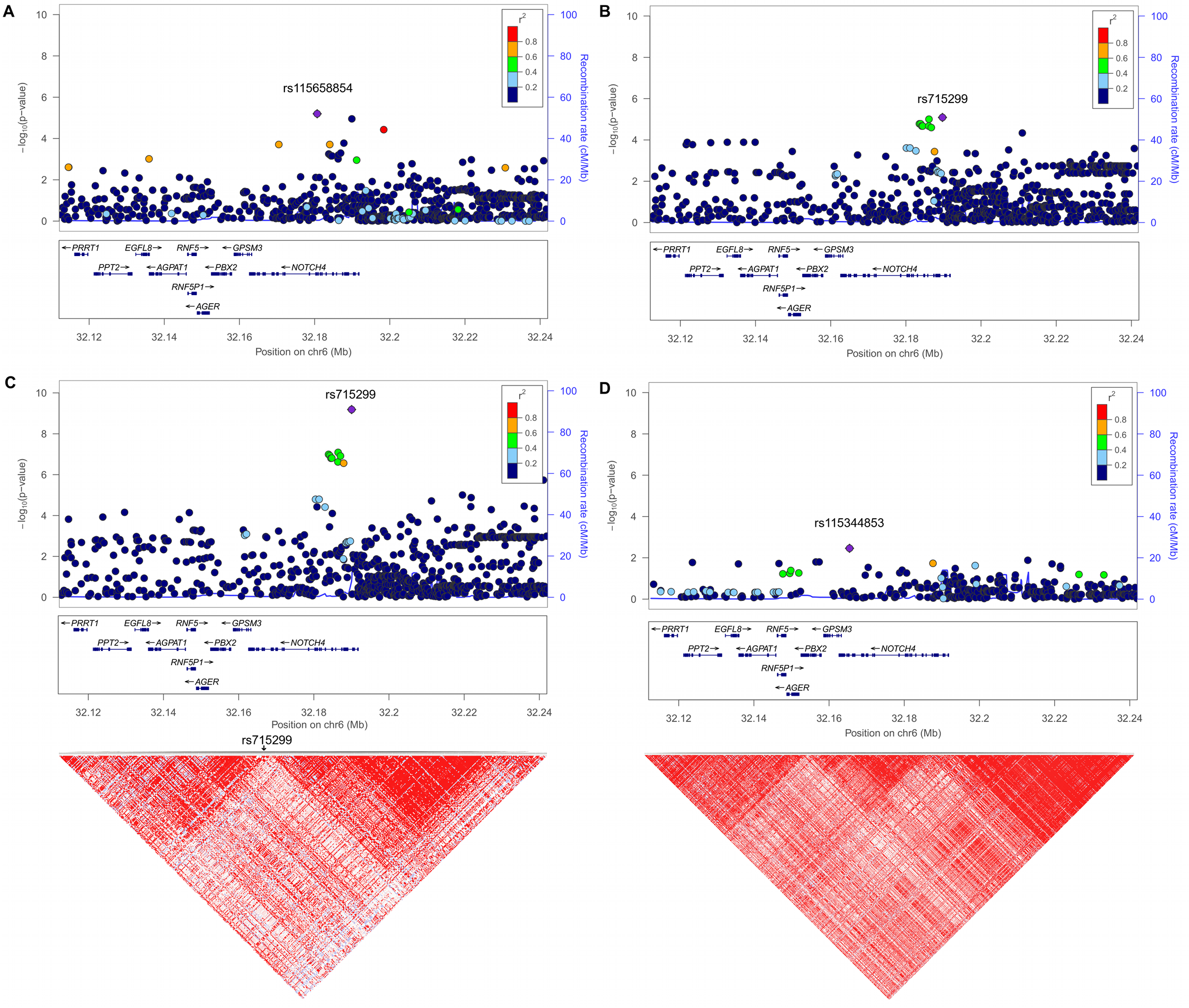


**Figure S3. Regional association plots of SNP-sarcoidosis association test results within *NOTCH4.***

(A-D) Association results in the AA discovery set (A), AA replication set (B), a meta-analysis between the AA discovery and AA replication sets including the LD (*D*’) plot (C), and the EA dataset including the LD (*D*’) plot (D). Each SNP is colored according to its LD (*r*^2^) with the top SNP. The blue solid line denotes the recombination rate.
