# Supplementary material for: Genome-Wide Association Study of African and European Americans Implicates Multiple Shared and Ethnic Specific Loci in Sarcoidosis Susceptibility
Source: PLoS One. 2012 Aug 27;7(8):e43907. doi: 10.1371/journal.pone.0043907 (PMC3428296; doi:10.1371/journal.pone.0043907)
Supplement: Table S1 — Summary of dropped samples after QC. (DOC) [file pone.0043907.s004.doc]

**Table S1. Summary of dropped samples after QC.**

| No. | Quality Control | # of AA Samples | # of EA Samples |
| --- | --- | --- | --- |
| 1 | Unknown Gender | 3 | 3 |
| 2 | Unknown Affection Status | 2 | 3 |
| 3 | Duplicates | 171 | 14 |
| 4 | Samples with Low Call Rate (<90%) | 72 | 50 |
| 5 | Heterozigosity outliers | 1 | 4 |
| 6 | Extreme population outliers | 6 | 87 |
| 7 | Cryptic relatedness | 0 | 124 |
| 8 | Not included in the 1:5 case-control matching | 0 | 1094 |
|  | Total Removed | 255 | 1379 |
