# Supplementary material for: Genome-Wide Association Study of African and European Americans Implicates Multiple Shared and Ethnic Specific Loci in Sarcoidosis Susceptibility
Source: PLoS One. 2012 Aug 27;7(8):e43907. doi: 10.1371/journal.pone.0043907 (PMC3428296; doi:10.1371/journal.pone.0043907)
Supplement: Table S6 — Shared or Ethnic Specific Suggestive Association Regions supported by the heterogeneity test results and list of inflammatory or lung diseases associated with these regions. (DOC) [file pone.0043907.s009.doc]

**Table S6**. Shared or Ethnic Specific Suggestive Association Regions supported by the heterogeneity test results and list of inflammatory or lung diseases associated with these regions.

| **CHR** | **BP(hg 19)** | **SNP** | **Gene** | **Alleles^1^** | **African Americans** | | | | | | |  | **European Americans** | | |  | **P_All-Meta_** | **Heteroge-neity Test** | | **Disease Related** |
| --- | --- | --- | --- | --- | --- | --- | --- | --- | --- | --- | --- | --- | --- | --- | --- | --- | --- | --- | --- | --- |
|  |  |  |  |  | **MAF_AA-Disc_^2^** | **OR_AA-Disc_** | ***P*_AA-Disc_** | **MAF_AA-Rep_^2^** | **OR_AA-Rep_** | ***P*_AA-Rep_** | **P_AA-Meta_** |  | **MAF_EA_^2^** | **OR_EA_** | ***P*_EA_** |  |  | ***Q*** | ***I^2^* (%)** |  |
| 3 | 42,174,148 | rs73066377^a^ | *TRAK1* | C/T | 0.410 | 1.050 | 6.89E-03 | 1.313 | 1.313 | 3.58E-03 | 9.46E-05 |  | 0.518 | 1.217 | 9.30E-03 |  | 4.06E-06 | 0.280 | 21.5 | N/A |
| 6 | 31,841,055 | rs9267655^a^ | *SLC44A4* | G/A | 0.257 | 0.760 | 9.23E-05 | 0.824 | 0.824 | 7.81E-02 | 2.70E-05 |  | 0.096 | 0.629 | 1.95E-03 |  | 2.37E-07 | 0.347 | 5.6 | N/A |
| 7 | 42,613,720 | rs7811252^a^ | *GLI3-C7orf25* | G/A | 0.398 | 0.764 | 1.77E-07 | 0.841 | 0.841 | 8.52E-02 | 1.66E-07 |  | 0.038 | 0.729 | 1.45E-01 |  | 1.78E-06 | 0.740 | 0 | N/A |
| 13 | 26,428,607 | rs9319250^a^ | *ATP8A2* | T/C | 0.061 | 1.641 | 1.28E-05 | 1.344 | 1.344 | 7.52E-02 | 4.72E-06 |  | 0.174 | 1.299 | 6.02E-03 |  | 2.08E-07 | 0.580 | 0 | N/A |
| 20 | 2,284,264 | rs214789^a^ | *TGM3* | G/A | 0.513 | 0.795 | 1.93E-05 | 0.847 | 0.847 | 8.83E-02 | 8.33E-06 |  | 0.183 | 0.782 | 1.72E-02 |  | 1.24E-06 | 0.836 | 0 | celiac disease , psoriasis |
| 3 | 60,872,879 | rs7613542^b^ | *FHIT* | C/T | 0.451 | 0.829 | 6.04E-05 | 0.849 | 0.849 | 7.62E-02 | 1.82E-05 |  | 0.222 | 1.140 | 1.38E-01 |  | 4.02E-02 | 0.026 | 72.6 | lung cancer |
| 6 | 106,588,806 | rs6568431^b^ | *PRDM1* | C/A | 0.441 | 0.835 | 1.40E-04 | 0.801 | 0.801 | 1.72E-02 | 7.42E-06 |  | 0.383 | 1.144 | 7.80E-02 |  | 4.58E-02 | 0.005 | 81.3 | rheumatoid arthritis , inflammatory bowel disease (IBD) , and SLE |
| 9 | 86,021,132 | rs7035287^b^ | *FRMD3* | C/T | 0.411 | 0.822 | 4.15E-04 | 0.727 | 0.727 | 1.09E-03 | 1.79E-06 |  | 0.621 | 1.040 | 6.19E-01 |  | 2.01E-03 | 0.012 | 77.2 | lung cancer |
| 10 | 124,393,339 | rs4752712^b^ | *DMBT1* | G/A | 0.216 | 0.917 | 1.19E-02 | 0.690 | 0.690 | 1.47E-03 | 9.41E-05 |  | 0.062 | 1.139 | 3.88E-01 |  | 2.73E-02 | 0.026 | 72.5 | ulcerative colitis , Crohn’s disease |
| 15 | 85,171,317 | rs17598114^b^ | *ZSCAN2-ALPK3* | C/T | 0.033 | 2.191 | 9.79E-04 | 1.728 | 1.728 | 2.23E-02 | 6.07E-05 |  | 0.282 | 1.040 | 6.34E-01 |  | 1.31E-03 | 0.012 | 77.6 | N/A |
| 2 | 202,052,754 | rs16836813^c^ | *CASP10* | C/T | 0.094 | 1.002 | 7.79E-01 | 0.730 | 0.730 | 4.06E-02 | 3.28E-01 |  | 0.038 | 1.989 | 2.89E-05 |  | 2.76E-02 | 3.70E-05 | 90.2 | autoimmune lymphoprolifera-tive syndrome and non-Hodgkin lymphoma |
| 3 | 25,568,512 | rs13099641^c^ | *RARB* | T/A | 0.100 | 0.926 | 6.75E-01 | 1.203 | 1.203 | 1.84E-01 | 6.54E-01 |  | 0.136 | 1.532 | 1.87E-05 |  | 9.22E-04 | 0.031 | 71.1 | pulmonary function |
| 6 | 31,558,702 | rs3130063^c^ | *NCR3* | C/T | 0.078 | 1.084 | 5.81E-01 | 1.018 | 1.018 | 9.18E-01 | 6.12E-01 |  | 0.110 | 1.553 | 6.74E-05 |  | 1.61E-03 | 0.066 | 63.2 | pulmonary function |

^1^Major/minor allele of AAs as the reference; ^2^Minor allele frequency; ^3^The odds ratio (OR) was calculated with respect to the minor allele of AAs.

^a^Associated in both AAs and EAs; ^b^Associated in AAs only; ^c^Associated in EAs only

**References:**

1. Alaedini A, Green PH (2008) Autoantibodies in celiac disease. Autoimmunity 41: 19-26.

2. Uemura N, Nakanishi Y, Kato H, Saito S, Nagino M, et al. (2009) Transglutaminase 3 as a prognostic biomarker in esophageal cancer revealed by proteomics. Int J Cancer 124: 2106-2115.

3. Mehul B, Bernard D, Brouard M, Delattre C, Schmidt R (2006) Influence of calcium on the proteolytic degradation of the calmodulin-like skin protein (calmodulin-like protein 5) in psoriatic epidermis. Exp Dermatol 15: 469-477.

4. Candi E, Oddi S, Paradisi A, Terrinoni A, Ranalli M, et al. (2002) Expression of transglutaminase 5 in normal and pathologic human epidermis. J Invest Dermatol 119: 670-677.

5. Cecener G, Tunca B, Egeli U, Karadag M, Vatan O, et al. (2008) Mutation analysis of the FHIT gene in bronchoscopic specimens from patients with suspected lung cancer. Tumori 94: 845-848.

6. Demopoulos K, Arvanitis DA, Vassilakis DA, Siafakas NM, Spandidos DA (2002) MYCL1, FHIT, SPARC, p16(INK4) and TP53 genes associated to lung cancer in idiopathic pulmonary fibrosis. J Cell Mol Med 6: 215-222.

7. Raychaudhuri S, Thomson BP, Remmers EF, Eyre S, Hinks A, et al. (2009) Genetic variants at CD28, PRDM1 and CD2/CD58 are associated with rheumatoid arthritis risk. Nat Genet 41: 1313-1318.

8. Barrett JC, Hansoul S, Nicolae DL, Cho JH, Duerr RH, et al. (2008) Genome-wide association defines more than 30 distinct susceptibility loci for Crohn's disease. Nat Genet 40: 955-962.

9. Anderson CA, Boucher G, Lees CW, Franke A, D'Amato M, et al. (2011) Meta-analysis identifies 29 additional ulcerative colitis risk loci, increasing the number of confirmed associations to 47. Nat Genet 43: 246-252.

10. Han JW, Zheng HF, Cui Y, Sun LD, Ye DQ, et al. (2009) Genome-wide association study in a Chinese Han population identifies nine new susceptibility loci for systemic lupus erythematosus. Nat Genet 41: 1234-1237.

11. Gateva V, Sandling JK, Hom G, Taylor KE, Chung SA, et al. (2009) A large-scale replication study identifies TNIP1, PRDM1, JAZF1, UHRF1BP1 and IL10 as risk loci for systemic lupus erythematosus. Nat Genet 41: 1228-1233.

12. Haase D, Meister M, Muley T, Hess J, Teurich S, et al. (2007) FRMD3, a novel putative tumour suppressor in NSCLC. Oncogene 26: 4464-4468.

13. Fukui H, Sekikawa A, Tanaka H, Fujimori Y, Katake Y, et al. (2011) DMBT1 is a novel gene induced by IL-22 in ulcerative colitis. Inflamm Bowel Dis 17: 1177-1188.

14. Rosenstiel P, Sina C, End C, Renner M, Lyer S, et al. (2007) Regulation of DMBT1 via NOD2 and TLR4 in intestinal epithelial cells modulates bacterial recognition and invasion. J Immunol 178: 8203-8211.

15. Renner M, Bergmann G, Krebs I, End C, Lyer S, et al. (2007) DMBT1 confers mucosal protection in vivo and a deletion variant is associated with Crohn's disease. Gastroenterology 133: 1499-1509.

16. Wang J, Zheng L, Lobito A, Chan FK, Dale J, et al. (1999) Inherited human Caspase 10 mutations underlie defective lymphocyte and dendritic cell apoptosis in autoimmune lymphoproliferative syndrome type II. Cell 98: 47-58.

17. Shin MS, Kim HS, Kang CS, Park WS, Kim SY, et al. (2002) Inactivating mutations of CASP10 gene in non-Hodgkin lymphomas. Blood 99: 4094-4099.

18. Soler Artigas M, Loth DW, Wain LV, Gharib SA, Obeidat M, et al. (2011) Genome-wide association and large-scale follow up identifies 16 new loci influencing lung function. Nature genetics 43: 1082-1090.
